# Surface Roughness and Color Stability of S-PRG Giomer Flowable Restoratives Versus Nanofill Resin Composites After Simulated Toothbrushing: An In Vitro Study

**DOI:** 10.3390/polym18212615

**Published:** 2026-10-22

**Authors:** Yasser Merdad, Razan Alharbi, Abeer Tashkandi, Renad Bakhaider, Asaad H. Doum, Mohammed A. Hanbazazah

**Affiliations:** 1 Department of Restorative Dentistry, Faculty of Dentistry, King Abdulaziz University, P.O. Box 80209, Jeddah 21589, Saudi Arabia; rnalharbe@kau.edu.sa (R.A.); btashkndee@kau.edu.sa (A.T.); 2 King Abdullah International Medical Research Center, Riyadh 11426, Saudi Arabia; bakhaiderr@ksau-hs.edu.sa; 3 Department of Restorative and Prosthetic Dental Sciences, King Saud bin Abdulaziz University for Health Sciences, Riyadh 11481, Saudi Arabia; 4 Ministry of the National Guard–Health Affairs, Riyadh 11426, Saudi Arabia; 5 Faculty of Dentistry, King Abdulaziz University, Jeddah 21589, Saudi Arabia; asaaddoum60@gmail.com (A.H.D.); mahanbazazah@live.com (M.A.H.)

**Keywords:** giomer, S-PRG filler, resin composite, nanofill, surface roughness, CIEDE2000, simulated toothbrushing, scanning electron microscopy, Beautifil, Filtek Supreme Ultra

## Abstract

This in vitro study compared surface roughness (Ra) and CIEDE2000 color change (ΔE00) of two giomer flowables with surface pre-reacted glass-ionomer (S-PRG) filler, Beautifil Flow Plus F00 and Beautifil Flow Plus X F00, and two nanofill resin composites, Filtek Supreme Ultra Flowable and the packable Filtek Supreme Ultra Universal, during simulated toothbrushing. Ten discs per material were cured against glass without finishing or polishing and brushed for 10,000 linear cycles at 2.5 N. Ra and color were measured at baseline and every 2500 cycles; scanning electron microscopy illustrated the surfaces. Beautifil Flow Plus X became smoother after the first 2500 cycles (*p* ≤ 0.001), and no material became significantly rougher. At 10,000 cycles, Filtek Supreme Ultra Universal was rougher than the three flowables. ΔE00 differed among materials (*p* < 0.001) but not between intervals; Beautifil Flow Plus X changed the least (mean 0.408), less than Beautifil Flow Plus (0.886) and Filtek Supreme Ultra Universal (0.768), and no disc reached the 50:50% acceptability threshold of 1.8. Without an unbrushed control, ΔE00 reflects brushing, storage, and measurement combined. Under this protocol, Beautifil Flow Plus X became smoother and changed color the least.

## 1. Introduction

Resin composites are considered the main direct restorative materials for anterior and posterior teeth [1]. Their widespread use is supported by adhesive placement, sufficient mechanical strength, and a wide range of available shades. However, surface degradation remains a limitation to clinical longevity. Toothbrush abrasion can increase surface roughness (Ra) by wearing the resin matrix and dislodging filler particles [2]. Increased roughness can promote plaque retention [3].

Filtek Supreme Ultra is a nanofilled composite in which silica and zirconia nanomers and nanoclusters are dispersed in a dimethacrylate matrix. This formulation was developed to improve polish retention and wear resistance compared with microhybrid composites [4]. These advantages have been generally supported by toothbrushing studies of nanofilled materials [2], although outcomes vary with the finishing and polishing protocol [5]. Packable, or high-viscosity, versions of the same chemistry contain a greater filler content than their flowable counterparts [6]. This increase in viscosity might influence abrasion resistance and might also increase the risk of void formation during placement.

Giomers are resin-based restoratives that contain surface pre-reacted glass-ionomer (S-PRG) fillers, in which the surface of a fluoro-boroaluminosilicate glass core is pre-reacted to form a glass-ionomer phase. Unlike conventional composites, giomers combine a resin matrix with ion-releasing fillers [7,8,9]. They release fluoride, strontium, borate, sodium, silicate, and aluminum ions and can be recharged with fluoride from external sources [10]. These ion-release properties have been associated with acid neutralization, mineralization-related effects, and other bioactive functions [10,11]. Beautifil Flow Plus X is a flowable giomer indicated for anterior and posterior restorations, including Class I–V cavities. According to the manufacturer, it combines the esthetic and mechanical properties of nanofilled resin composites with the bioactive potential of S-PRG fillers, including fluoride release and recharge [12]. Although giomers have shown adequate mechanical properties [13], Beautifil Flow Plus and the submicron-filled Beautifil Flow Plus X have not been compared with nanofilled flowable and packable composites under a standardized toothbrushing protocol. Whether the two giomers, which differ in S-PRG particle size, also differ in color stability under such a protocol has not been reported.

Both packable and flowable resin composites are exposed to mechanical challenges during routine toothbrushing. Simulated brushing can result in higher surface roughness and less gloss, and the degree of deterioration depends on material composition and filler characteristics [14,15]. Furthermore, abrasion can change the surface morphology of bioactive flowable composites and promote filler exposure or dislodgement [16]. Such modifications may affect the long-term esthetic and functional performance of restorations [17,18]. Ra values above 0.2 µm have been linked to increased plaque accumulation and, through it, a greater risk of caries and periodontal inflammation [3].

Miyano et al. included Beautifil Flow Plus and Beautifil Flow Plus X in a toothbrush-abrasion study of flowable composites, but that work focused on abrasion volume and surface morphology. It did not assess optical color change or compare these two S-PRG giomers with both a nanofilled flowable and a nanofilled packable composite under the same experimental conditions [16]. This in vitro study therefore compared four commercial products during 10,000 simulated toothbrushing cycles: the giomer flowables Beautifil Flow Plus and Beautifil Flow Plus X, the nanofilled flowable Filtek Supreme Ultra Flowable, and the nanofilled packable Filtek Supreme Ultra Universal. That cycle count is a laboratory convention often taken to approximate one year of twice-daily brushing [19,20]. Surface roughness was evaluated using Ra, color change was quantified using the CIEDE2000 color difference (ΔE00), and scanning electron microscopy was used to illustrate surface morphology. The null hypotheses were that (i) Ra would not differ among the materials or across the brushing intervals, and (ii) ΔE00 would not differ among the materials or across the brushing intervals.

## 2. Materials and Methods

### 2.1. Study Design and Sample Size

This in vitro study evaluated 4 resin composite materials for surface roughness and color stability after simulated toothbrushing. The materials were examined at baseline and after 2500, 5000, 7500, and 10,000 brushing cycles. Ten disc specimens were analyzed per material (N = 40).

### 2.2. Materials

Table 1 presents the four restoratives: Beautifil Flow Plus F00 (Shofu Inc., Kyoto, Japan), Beautifil Flow Plus X F00 (Shofu, San Marcos, CA, USA), Filtek Supreme Ultra Flowable Restorative (3M Oral Care, St. Paul, MN, USA), and Filtek Supreme Ultra Universal Restorative (packable/body; 3M Oral Care, St. Paul, MN, USA). All specimens were shade A2. Materials were stored and used within their expiry dates. The compositions in Table 1 are manufacturer-provided values [6,21].

### 2.3. Specimen Preparation

Forty disc-shaped composite specimens were fabricated using a standardized mold with an inner diameter of 8 mm and a height of 1.5 mm, *n* = 10 per material (N = 40). The mold was secured between two glass plates with clips so that excess material was extruded. Disc diameter was chosen to fully accommodate the 6 mm spectrophotometer aperture. Each specimen was polymerized from the top surface for 20 s in standard mode with a multi-wave light-emitting diode (LED) curing unit (VALO Cordless, Ultradent, South Jordan, UT, USA), according to the manufacturer’s instructions. After light curing, specimens were inspected for visible voids; discs with imperfections were excluded. The top surface of each disc, which was later brushed, measured, and imaged, was left as cured against the glass plate and was not finished or polished. All four materials were prepared in the same way. Before baseline readings, specimens were stored for 24 h in distilled water in a sealed glass container. All procedures were performed by a single clinician. Mean disc thickness was 1.52 ± 0.02 mm (range 1.47–1.54 mm).

### 2.4. Surface Roughness Measurement

Ra of each specimen was recorded with a contact profilometer (Surftest SJ-210, Mitutoyo, Kawasaki, Japan) in accordance with ISO 4287:1997 [22]. The instrument was set to a 0.8 mm cut-off length and a 0.5 mm/s scanning speed. The diamond stylus had a 5 µm tip radius, a 90° cone angle, and a measuring force of 4 mN. Measurements were made at baseline and after each 2500-cycle interval up to 10,000 cycles. Three consecutive roughness traces were collected from the top surface of each disc. The mean of the three traces was the specimen Ra for that interval.

### 2.5. Color Measurement

Color measurements were obtained using a spectrophotometer (CM-700d, Konica Minolta, Tokyo, Japan) according to the CIE L*a*b* coordinate system. The instrument has a diffuse-illumination, 8° viewing (d:8) sphere geometry and was equipped with a 6 mm aperture; calibration with white and black tiles was performed before each session. The illuminant, standard observer, and specular-component settings were not recorded. The same instrument, aperture, backing, and settings were used at every interval, so each ΔE00 value is a within-disc difference measured under identical conditions. Specimens were positioned on a 50% gray background (L*: 51.44, a*: −0.92, b*: −6.38) during measurements. Baseline color records for all specimens were taken prior to brushing and used as references for subsequent evaluations. Each disc was read three times, and the readings were averaged to give its L*, a*, and b* values. The readings were repeated after 2500, 5000, 7500, and 10,000 brushing cycles. The color change from baseline at each interval, ΔE00, was calculated with the CIEDE2000 formula of Luo et al. as implemented by Sharma et al., with k_L_ = k_C_ = k_H_ = 1 [23,24,25]:

ΔE_00_ = [(ΔL′/(k_L_·S_L_))^2^ + (ΔC′/(k_C_·S_C_))^2^ + (ΔH′/(k_H_·S_H_))^2^ + R_T_·(ΔC′/(k_C_·S_C_))·(ΔH′/(k_H_·S_H_))]^1/2^

In this equation, ΔL′, ΔC′, and ΔH′ are the differences in lightness, chroma, and hue, respectively. R_T_ is a rotation function that accounts for the interaction between chroma and hue differences in the blue region. S_L_, S_C_, and S_H_ are weighting functions that compensate for non-uniformities in the color space. The parametric factors k_L_, k_C_, and k_H_ were set to 1.

### 2.6. Toothbrushing Simulation

After baseline Ra and color measurements, specimens underwent simulated toothbrushing in a multi-station chewing simulator (SD Mechatronik, Feldkirchen-Westerham, Germany). A custom mold and holder for the simulator were fabricated with a Snapmaker U1 3D printer (Snapmaker, Shenzhen, China) to facilitate specimen preparation and testing. The specimens were then mounted onto the simulator’s test stations.

The slurry was one part Colgate Fresh Confidence Gel toothpaste (Colgate-Palmolive, New York, NY, USA) [26] to three parts deionized water by volume, and the discs stayed submerged in it throughout brushing. Soft-bristled toothbrushes (Tara, Dammam, Saudi Arabia) [27] were mounted on the simulator under a constant load of 2.5 N. The height and angulation of each toothbrush were adjusted to maintain consistent contact between the brush head and specimen surface. Toothbrushes were replaced when the bristles showed visible signs of wear. The device operated in a linear motion over an 8 mm stroke at a speed of 40 mm/s for a total of 10,000 brushing cycles; one cycle was defined as one forward and one backward stroke (two passes). The simulator had four stations, so four discs were brushed at the same time, one per station, and each disc stayed in the same station for all 10,000 cycles. The slurry was renewed at every 2500-cycle interval. Ten thousand linear cycles were considered a laboratory analogue for approximately one year of twice-daily brushing, consistent with previous dental materials protocols [19,20].

At every 2500-cycle interval, the acrylic blocks were removed, thoroughly rinsed under running water, and the specimens were detached and air-dried. The drying time before measurement was not recorded. Color and surface roughness were measured at each interval.

### 2.7. Scanning Electron Microscopy Imaging

Scanning electron microscopy (SEM) was used to illustrate surface morphology. One additional disc of each material was prepared in the same way as the test discs and reserved for SEM before brushing; it was not one of the 40 discs used for the Ra and color measurements. The samples were cleaned in an ultrasonic cleaner and, to improve surface conductivity and imaging resolution, covered with a thin layer of gold using a sputter coater before imaging. Each SEM disc was imaged at baseline, brushed for 10,000 cycles alongside the test discs with the baseline gold coating left in place, then cleaned, coated again, and imaged a second time. Because the baseline coating remained on the surface during brushing, these discs were excluded from the quantitative analysis. The coated samples were examined using a scanning electron microscope (AURA100, Seron Technologies Inc., Uiwang-si, Republic of Korea) at magnifications of ×50, ×100, ×500, ×1000, ×1500, and ×2000. The SEM operated at an accelerating voltage of 10 kV with a working distance of approximately 9.5 mm. The images are illustrative and were not quantified.

### 2.8. Statistical Analysis

Each disc was the unit of analysis; its three Ra traces and three color readings were averaged at each interval, and data are presented as mean ± standard deviation (SD). Ra and ΔE00 were each analyzed with a two-way repeated-measures design (material × brushing interval) using a linear mixed-effects model with main effects and interaction. The model treated each disc as its own unit (a random intercept per disc) and allowed each material its own amount of scatter between discs, because the packable composite varied far more than the flowables; a likelihood-ratio test confirmed that this fitted the data better than one common scatter. The model was fitted by restricted maximum likelihood. A simple effects analysis was used to interpret the interaction where it was significant, and pairwise comparisons were used to interpret main effects. Pairwise comparisons were made on the model estimates (Satterthwaite degrees of freedom), Holm-corrected, and reported with unadjusted 95% confidence intervals (CIs). Three checks were run on each model: the residuals were examined with plots and the Shapiro–Wilk test; the model was refitted 40 times, leaving out one disc each time, to see whether any single disc drove the results; and, where the residuals were not normally distributed, the analysis was repeated on log10-transformed values. Statistical analysis was performed with R (version 4.3.1; packages nlme 3.1-162 and emmeans 1.11.2; R Foundation for Statistical Computing, Vienna, Austria) and JASP (version 0.98.1; JASP Team, University of Amsterdam, Amsterdam, The Netherlands), and *p* < 0.05 was considered statistically significant.

## 3. Results

### 3.1. Surface Roughness

**Figure 1:**
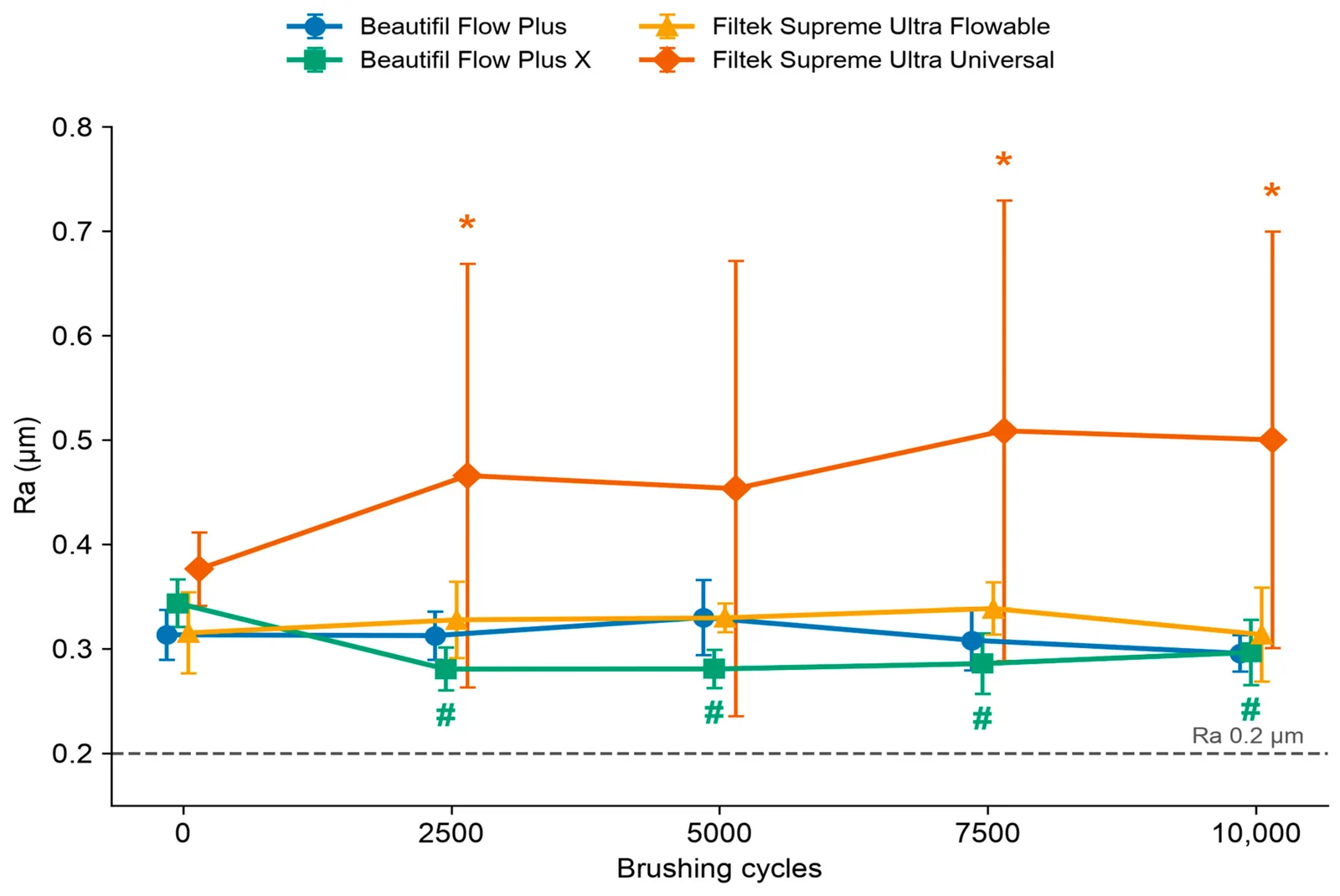
Mean Ra (± SD) as a function of brushing cycles. # Beautifil Flow Plus X has a lower Ra than its own baseline (Holm-adjusted *p* ≤ 0.001). * Filtek Supreme Ultra Universal is rougher than all three flowables (Holm-adjusted *p* ≤ 0.049). Its large SD reflects large differences between discs. The dashed line marks the 0.2 µm plaque-retention threshold reported by Bollen et al. [3]. Symbols are offset horizontally for clarity; all pairwise comparisons are given in Table 2.

The two-way repeated-measures analysis of Ra showed no significant effect of brushing interval (F(4, 144) = 0.32, *p* = 0.862), a significant main effect of material (F(3, 36) = 13.41, *p* < 0.001), and a significant material × brushing interval interaction (F(12, 144) = 5.66, *p* < 0.001) (Table 2 and Figure 1). Because the interaction was significant, simple effects were analyzed and followed by pairwise comparisons.

The materials did not differ at baseline (simple effect of material, *p* = 0.059) but differed at every brushing interval (*p* ≤ 0.007). Pairwise comparisons (lowercase letters in Table 2) showed that, after brushing, Filtek Supreme Ultra Universal had the highest mean Ra at every interval. It was significantly rougher than all three flowables at 2500 cycles (*p* ≤ 0.049), 7500 cycles (*p* ≤ 0.018), and 10,000 cycles (*p* ≤ 0.011); at 5000 cycles, the difference was significant only against Beautifil Flow Plus X (*p* = 0.021) and not against Beautifil Flow Plus or Filtek Supreme Ultra Flowable (*p* = 0.123 for both), as its discs varied widely at that interval. At 10,000 cycles, it was rougher than Beautifil Flow Plus by 0.204 µm (95% CI 0.086 to 0.323), Beautifil Flow Plus X by 0.204 µm (95% CI 0.085 to 0.322), and Filtek Supreme Ultra Flowable by 0.187 µm (95% CI 0.068 to 0.305), and the three flowables did not differ from one another (*p* ≥ 0.538). Among the flowables, Beautifil Flow Plus X was significantly smoother than Beautifil Flow Plus (*p* = 0.049 at 2500 cycles; *p* = 0.001 at 5000 cycles) and Filtek Supreme Ultra Flowable (*p* = 0.004 and *p* = 0.002), and Filtek Supreme Ultra Flowable was significantly rougher than Beautifil Flow Plus (*p* = 0.0495) and Beautifil Flow Plus X (*p* = 0.001) at 7500 cycles.

The simple effect of brushing interval was significant for Beautifil Flow Plus X (*p* < 0.001) and Beautifil Flow Plus (*p* = 0.024) but not for Filtek Supreme Ultra Flowable (*p* = 0.156) or Filtek Supreme Ultra Universal (*p* = 0.514). Pairwise comparisons (uppercase letters in Table 2) showed that Beautifil Flow Plus X became smoother: its Ra was lower than at baseline at every interval, by 0.047 to 0.063 µm (Holm-corrected *p* ≤ 0.001; at 10,000 cycles, −0.047 µm, 95% CI −0.066 to −0.028). Beautifil Flow Plus did not differ from baseline at any interval (*p* ≥ 0.623); its only change was a 0.034 µm decrease between 5000 and 10,000 cycles (*p* = 0.012). The change of Filtek Supreme Ultra Universal at 10,000 cycles was +0.124 µm (95% CI −0.041 to 0.289, *p* = 1.000).

After brushing, Filtek Supreme Ultra Universal varied much more between discs than the flowables did (SD 0.200–0.220 µm versus 0.045 µm or less). Mean Ra of every material, including at baseline, was above 0.2 µm.

No single disc drove these results. When the model was refitted without each disc in turn, the material × brushing interval interaction stayed significant in all 40 refits (*p* < 0.001), and Filtek Supreme Ultra Universal stayed rougher than each flowable at 10,000 cycles (Holm-corrected *p* ≤ 0.049). The residuals of the Ra model departed mildly from normality (Shapiro–Wilk W = 0.981, *p* = 0.008; one of 200 residuals beyond ±3), so the analysis was repeated on log10-transformed Ra; the residuals were then normally distributed (W = 0.998, *p* = 0.996), and every conclusion was the same.

### 3.2. Color Difference (ΔE00)

**Figure 2:**
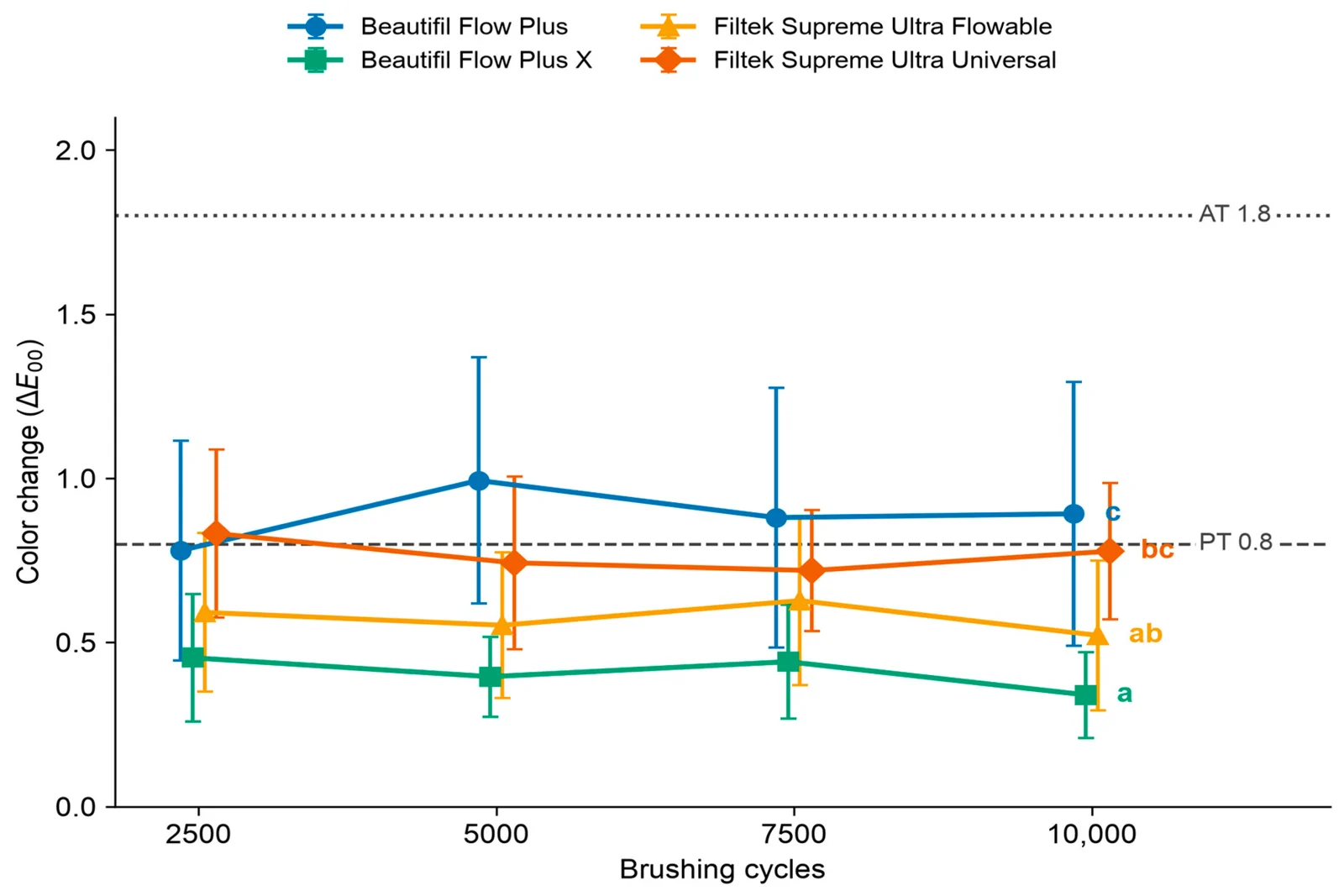
Mean ΔE00 (± SD) versus baseline. Letters on the right: materials that share no letter differ significantly over all intervals (Holm-adjusted *p* ≤ 0.012). Dashed line: 50:50% perceptibility threshold (PT, 0.8); dotted line: 50:50% acceptability threshold (AT, 1.8) [28,29]. Symbols are offset horizontally for clarity.

The two-way repeated-measures analysis of ΔE00 showed no significant effect of brushing interval (F(3, 108) = 0.48, *p* = 0.699), a significant main effect of material (F(3, 36) = 9.69, *p* < 0.001), and no significant material × brushing interval interaction (F(9, 108) = 1.42, *p* = 0.189) (Table 3 and Figure 2). Because the interaction was not significant, the main effect of material was followed by pairwise comparisons; intervals were not compared because the effect of brushing interval was not significant.

Averaged over the four intervals, mean ΔE00 was 0.408 for Beautifil Flow Plus X, 0.573 for Filtek Supreme Ultra Flowable, 0.768 for Filtek Supreme Ultra Universal, and 0.886 for Beautifil Flow Plus. Pairwise comparisons (letters in Table 3) showed that Beautifil Flow Plus X changed less than Beautifil Flow Plus (difference 0.479, 95% CI 0.282 to 0.676, *p* < 0.001) and Filtek Supreme Ultra Universal (0.360, 95% CI 0.169 to 0.551, *p* = 0.003), and that Filtek Supreme Ultra Flowable changed less than Beautifil Flow Plus (0.313, 95% CI 0.113 to 0.513, *p* = 0.012); Filtek Supreme Ultra Flowable did not differ significantly from Beautifil Flow Plus X (*p* = 0.171) or Filtek Supreme Ultra Universal (*p* = 0.149), nor did Filtek Supreme Ultra Universal from Beautifil Flow Plus (*p* = 0.239). At 10,000 cycles, mean ΔE00 was 0.340 (95% CI 0.247 to 0.434) for Beautifil Flow Plus X, 0.521 (0.358 to 0.684) for Filtek Supreme Ultra Flowable, 0.778 (0.629 to 0.927) for Filtek Supreme Ultra Universal, and 0.892 (0.604 to 1.179) for Beautifil Flow Plus. No Beautifil Flow Plus X disc exceeded the 50:50% perceptibility threshold (PT) of 0.8 at any interval, whereas 6 of 10 Beautifil Flow Plus discs exceeded it at 10,000 cycles. None of the 160 post-baseline ΔE00 values reached the 50:50% acceptability threshold (AT) of 1.8; the largest was 1.73 (one Beautifil Flow Plus disc at 10,000 cycles).

The residuals of the ΔE00 model were normally distributed (Shapiro–Wilk W = 0.989, *p* = 0.240), and refitting the model without each disc in turn did not change the significance of any comparison.

### 3.3. Scanning Electron Microscopy

**Figure 3:**
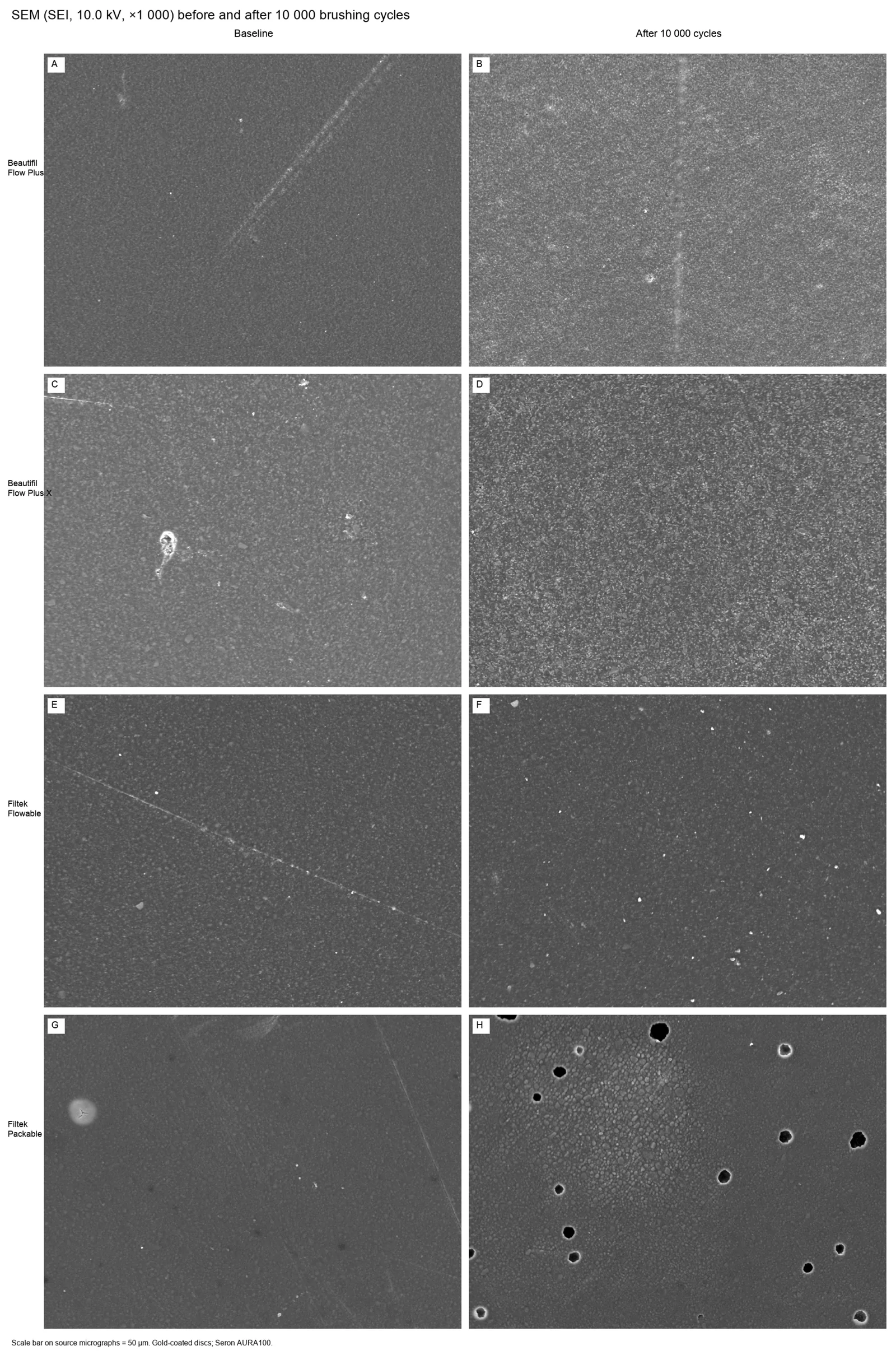
Secondary-electron SEM images (10.0 kV, ×1000; scale bar 50 µm) at baseline (**A**,**C**,**E**,**G**) and after 10,000 brushing cycles (**B**,**D**,**F**,**H**). (**A**,**B**) Beautifil Flow Plus; (**C**,**D**) Beautifil Flow Plus X; (**E**,**F**) Filtek Supreme Ultra Flowable; (**G**,**H**) Filtek Supreme Ultra Universal (packable). Panel H shows circular voids and exposed filler clusters on the brushed Filtek Supreme Ultra Universal disc. The images are illustrative and come from one representative disc of each material.

In the imaged discs, baseline surfaces of the three flowables were relatively homogeneous at ×1000, with occasional fine scratches (Figure 3A,C,E). After 10,000 cycles, the same materials showed a finely abraded texture. Beautifil Flow Plus showed a linear brushing track. No filler plucking or cavitation was seen on the flowable surfaces (Figure 3B,D,F). Beautifil Flow Plus X after brushing displayed a granular, uniformly exposed submicron filler field.

The imaged packable nanofill differed. Baseline Filtek Supreme Ultra Universal was smooth aside from isolated surface defects (Figure 3G). After 10,000 cycles, its surface showed circular voids, from a few micrometers to tens of micrometers, and islands of exposed filler clusters (Figure 3H). That morphology was not observed on the imaged flowable surfaces.

## 4. Discussion

We tested whether Ra and ΔE00 differed among four restoratives during 10,000 linear brushing cycles. The first null hypothesis was rejected. Ra differed among the materials, and the materials changed differently over the cycles: Beautifil Flow Plus X became smoother, whereas the other three materials showed no significant change from baseline. The second null hypothesis was partially rejected: ΔE00 differed among the materials but did not change significantly across the brushing intervals.

The interaction reflected different patterns over time, not uniform roughening of one material. Beautifil Flow Plus X became smoother within the first 2500 cycles and stayed smoother than its baseline, whereas Filtek Supreme Ultra Universal varied widely between discs and did not change significantly overall. Beautifil Flow Plus and Filtek Supreme Ultra Flowable did not differ from their baseline, and at 10,000 cycles the three flowables did not differ significantly from one another (Table 2). However, the study was not designed to test equivalence. A non-significant change therefore does not show that a material is stable. Baseline Ra of all four materials was already above 0.2 µm on these glass-cured surfaces, so this study measured how brushing changed a surface that was already above that value, not whether brushing pushed a smooth surface past it. The 0.2 µm value is a threshold for bacterial plaque retention derived from in vivo studies [3]. The clinical importance of changes of this size remains unknown.

AlAli et al. reported that Ra of three resin composites increased after 5000 brushing cycles [18], and Oliveira et al. reported that most composites, including Filtek Z350, became rougher than their own baseline after 100,000 strokes [2]. In contrast, Komalsingsakul et al. found no significant difference in roughness between brushed and unbrushed Beautifil II or Filtek Z350 XT after 20,000 strokes [30]. Our profilometric results are consistent with the SEM findings. The imaged flowable surfaces showed a finely abraded texture without filler plucking, and Beautifil Flow Plus X showed a uniformly exposed submicron filler field, in line with its lower Ra after brushing. The brushed Filtek Supreme Ultra Universal surface was different, with circular voids and exposed filler clusters. This packable material is more viscous than the flowables, is packed rather than injected, and has a higher filler loading that includes zirconia/silica nanoclusters [4,6]. A heavier consistency does not by itself make a composite stronger; in packable composites, mechanical properties followed resin chemistry and filler volume [31]. Isolated defects were already seen on the baseline SEM image of this material, so brushing may have uncovered small pores near the surface, introduced when the high-viscosity paste was packed into the mold, rather than created them. The SEM images are illustrative and do not prove this mechanism. Because all discs were prepared in the same way, these results describe each product as it was placed, not the product alone.

The 50:50% perceptibility threshold (ΔE00 = 0.8) is the color difference that half of observers can notice, and the 50:50% acceptability threshold (ΔE00 = 1.8) is the difference that half of observers judge unacceptable [28,29]. No group mean or individual ΔE00 value reached 1.8. The differences between materials are therefore laboratory differences measured under one protocol, and their clinical relevance is unknown. Within that range, Beautifil Flow Plus X showed the smallest ΔE00 at every interval, and none of its discs exceeded 0.8. Beautifil Flow Plus accounted for most of the discs whose change was perceptible but still acceptable, and its ΔE00 was also larger than that of Filtek Supreme Ultra Flowable. The larger ΔE00 of Beautifil Flow Plus than of Beautifil Flow Plus X is unlikely to come from surface roughness alone, because the Ra of the two materials did not differ significantly at 10,000 cycles (Table 2). A giomer stored in water for 28 days showed greater water sorption and color change than two nanohybrid composites [32], and composite staining has been shown to depend more on monomer and filler composition than on surface smoothness [33]. The two Beautifil materials differ in monomer composition, S-PRG particle size, and filler loading (Table 1). Because these factors vary together between commercial products, we could not identify which of them caused the color difference.

Discoloration of tooth-colored resin-based materials can result from intrinsic and extrinsic factors. Intrinsic discoloration involves changes within the resin matrix and at the matrix–filler interface, while extrinsic discoloration results from the adsorption or absorption of external colorants, such as those in coffee, tea, other beverages, and nicotine [33]. No unbrushed control was stored and measured on the same schedule in this study. The ΔE00 values therefore describe the combined effect of brushing, water storage, rinsing, drying, and repeated measurement and cannot be attributed to abrasion alone. Because ΔE00 did not change significantly across the intervals, most of the color change was already present at the first reading after baseline (2500 cycles), but its cause was not determined. The discs were stored in distilled water, not a staining solution, so progressive staining was not expected.

This in vitro study did not include a staining challenge, so these ΔE00 values do not describe resistance to coffee, wine, or chlorhexidine. Water uptake may contribute: during water storage, the giomer with the highest water sorption also discolored the most, and solubility correlated with color change [32]. Color stability appears to depend on several material-related factors, including resin-matrix and filler composition and surface characteristics, rather than on surface roughness alone. The between-material differences remain valid comparisons under the same combined protocol.

This study has several limitations. First, the simulator used one dentifrice, one brush type, and a linear stroke at a constant load. Brushing in the mouth is elliptical, takes place in saliva rather than deionized water, and is interrupted by dietary acids. In a comparison of six laboratory wear methods with clinical wear of composite resins, only one correlated with the clinical results [34]. The conversion of 10,000 cycles to one year is a laboratory convention [19,20], not a validated physiological equivalent; other studies have equated the same number of cycles with two years of brushing [15]. Second, no unbrushed control group was stored and measured over the same period, so the color change caused by brushing alone may be smaller than the ΔE00 reported here. Third, the four commercial products differ at the same time in resin matrix, filler chemistry, filler size and loading, and viscosity; the design therefore compares products, and we cannot attribute differences to S-PRG chemistry, filler size, or viscosity alone. Fourth, SEM was performed on one disc per material, so the images may not represent every disc in a group. Fifth, brushes were replaced when the bristles showed visible wear rather than on a fixed schedule, so discs may have been brushed by bristles at slightly different stages of wear. Gloss, fluoride release, and recharge were not measured. Findings should not be generalized to other giomers, other nanofills, or to finished or polished surfaces.

Under this protocol, no material became significantly rougher than its baseline, and Beautifil Flow Plus X both became smoother and showed the smallest ΔE00, smaller than that of Beautifil Flow Plus and Filtek Supreme Ultra Universal. The packable Filtek Supreme Ultra Universal was rougher than the flowables at 10,000 cycles, with large differences between discs. A randomized clinical comparison of Beautifil Flow Plus X and a nanofilled flowable, with color measured over at least one year, would show whether these laboratory differences persist in the mouth.

## 5. Conclusions

Within the limitations of this in vitro study, the following conclusions were drawn for discs cured against glass without finishing or polishing, after 10,000 cycles of linear toothbrushing in a 1:3 toothpaste slurry at 2.5 N:

1. No material became significantly rougher after brushing; Beautifil Flow Plus X became smoother after the first 2500 cycles. At 10,000 cycles, Filtek Supreme Ultra Universal was rougher than the three flowables.
2. Mean ΔE00 of all four materials, as well as every individual value, remained below the 50:50% acceptability threshold. Under the combined brushing, storage, and measurement protocol, Beautifil Flow Plus X showed a smaller ΔE00 than Beautifil Flow Plus and Filtek Supreme Ultra Universal, and none of its discs exceeded the perceptibility threshold.
3. Because no unbrushed control was included, the color change cannot be attributed to brushing alone.

## Figures and Tables

**Table 1:** Material composition [6,21].

Material	Manufacturer	Category	Resin/Filler (wt%)	Consistency
Beautifil Flow Plus F00	Shofu	Giomer, S-PRG	Bis-GMA, TEGDMA; S-PRG fluoro-boroaluminosilicate glass (~67.8 wt%)	Zero flow (F00)
Beautifil Flow Plus X F00	Shofu	Giomer, submicron S-PRG	Bis-GMA, TEGDMA, Bis-MPEPP; S-PRG (~63.7 wt%; ~0.4 µm)	Zero-flow (F00)
Filtek Supreme Ultra Flowable	3M	Nanofill composite	Bis-GMA, TEGDMA, substituted dimethacrylate; silica, zirconia, YbF3 (~62–65 wt%)	Flowable
Filtek Supreme Ultra Universal	3M	Nanofill composite	Bis-GMA, Bis-EMA, UDMA, TEGDMA, PEGDMA; silica nanomers and zirconia/silica nanoclusters (~74.5–78.5 wt%)	Packable

**Table 2:** Mean ± standard deviation of Ra (µm); *n* = 10 discs per material.

Material	Baseline	2500	5000	7500	10,000
Beautifil Flow Plus	0.313 ± 0.024 ^AB,a^	0.312 ± 0.023 ^AB,b^	0.330 ± 0.036 ^A,b^	0.308 ± 0.029 ^AB,a^	0.295 ± 0.018 ^B,a^
Beautifil Flow Plus X	0.343 ± 0.023 ^A,a^	0.280 ± 0.020 ^B,a^	0.280 ± 0.018 ^B,a^	0.286 ± 0.029 ^B,a^	0.296 ± 0.031 ^B,a^
Filtek Supreme Ultra Flowable	0.315 ± 0.039 ^A,a^	0.328 ± 0.036 ^A,b^	0.329 ± 0.014 ^A,b^	0.338 ± 0.025 ^A,b^	0.313 ± 0.045 ^A,a^
Filtek Supreme Ultra Universal	0.376 ± 0.035 ^A,a^	0.466 ± 0.203 ^A,c^	0.453 ± 0.218 ^A,b^	0.509 ± 0.220 ^A,c^	0.500 ± 0.200 ^A,b^

Values are mean ± SD (µm); *n* = 10 discs per material. Lowercase letters compare materials within a column, and uppercase letters compare intervals within a row; values that share no letter differ significantly (simple main effects followed by Holm-adjusted pairwise comparisons, *p* < 0.05).

**Table 3:** Mean ± SD of ΔE00 versus baseline and number of discs exceeding the 50:50% perceptibility (PT = 0.8) and acceptability (AT = 1.8) thresholds; *n* = 10.

Material	2500 (*n* > PT/AT)	5000	7500	10,000
Beautifil Flow Plus ^c^	0.780 ± 0.335 (4/0)	0.993 ± 0.375 (8/0)	0.880 ± 0.395 (7/0)	0.892 ± 0.402 (6/0)
Beautifil Flow Plus X ^a^	0.453 ± 0.194 (0/0)	0.396 ± 0.122 (0/0)	0.441 ± 0.173 (0/0)	0.340 ± 0.131 (0/0)
Filtek Supreme Ultra Flowable ^ab^	0.591 ± 0.242 (1/0)	0.552 ± 0.222 (2/0)	0.627 ± 0.257 (2/0)	0.521 ± 0.228 (1/0)
Filtek Supreme Ultra Universal ^bc^	0.832 ± 0.255 (4/0)	0.743 ± 0.263 (4/0)	0.719 ± 0.184 (2/0)	0.778 ± 0.208 (4/0)

The material × interval interaction and the interval effect were not significant, so materials were compared over all intervals: materials whose names share no superscript letter differ significantly (Holm-adjusted *p* < 0.05). (*n* > PT/AT): number of discs above the perceptibility and acceptability thresholds.

## Data Availability

The original contributions presented in this study are included in the article. Further inquiries can be directed to the corresponding author.

## Abbreviations

The following abbreviations are used in this manuscript:

S-PRG	Surface pre-reacted glass ionomer
Ra	Arithmetic mean surface roughness
ΔE00	CIEDE2000 color difference
SEM	Scanning electron microscopy
PT	50:50% perceptibility threshold (ΔE00 = 0.8)
AT	50:50% acceptability threshold (ΔE00 = 1.8)
CIE	Commission Internationale de l’Éclairage
CI	Confidence interval
